# Influence of the Magnetic Field Strength on Image Contrast in Gd-EOB-DTPA-enhanced MR Imaging: Comparison between 1.5T and 3.0T

**DOI:** 10.2463/mrms.mp.2015-0158

**Published:** 2016-04-28

**Authors:** Hirofumi Hata, Yusuke Inoue, Ai Nakajima, Shotaro Komi, Hiroki Miyatake

**Affiliations:** 1 Department of Radiology, Kitasato University Hospital; 2 Department of Diagnostic Radiology, Kitasato University School of Medicine, 1-15-1 Kitasato, Minami-ku, Sagamihara, Kanagawa 252-0374, Japan

**Keywords:** Gd-EOB-DTPA, magnetic field strength, quantitative evaluation, image contrast, contrast enhancement

## Abstract

**Purpose:**

We quantitatively investigated hepatic enhancement in gadolinium-ethoxybenzyl-diethylenetriamine pentaacetic acid (Gd-EOB-DTPA)-enhanced magnetic resonance (MR) imaging at 1.5T and 3.0T.

**Methods:**

A total of 40 patients who underwent Gd-EOB-DTPA-enhanced MR imaging were included in the study. Precontrast and hepatobiliary-phase images acquired at a low flip angle (FA, 12°) and hepatobiliary-phase images acquired at a high FA (30°) were analyzed. From these images, the liver-to-muscle signal intensity ratio (LMR) and liver-to-spleen signal intensity ratio (LSR) were estimated, and the contrast enhancement ratio (CER) was calculated from the liver signal, LMR, and LSR as the ratio of the low-FA hepatobiliary-phase value to the precontrast value. The coefficient of variance in the liver signal was determined to represent image noise.

**Results:**

LMR and LSR indicated similar image contrast between 1.5T and 3.0T. A higher FA provided larger LMRs and LSRs, and the degree of the FA-dependent increase was similar between 1.5T and 3.0T. CER did not differ significantly between 1.5T and 3.0T, regardless of the calculation method. A better correlation to CER calculated from the liver signal was found for the LMR-based CER values than for the LSR-based CER. The coefficient of variance in the liver signal was significantly smaller at 3.0T for precontrast and low-FA hepatobiliary-phase images, but not for high-FA hepatobiliary-phase images.

**Conclusion:**

The indices of hepatic enhancement were similar between 1.5T and 3.0T, indicating that the magnetic field strength does not substantially influence image contrast after administration of Gd-EOB-DTPA.

## Introduction

Gadolinium-ethoxybenzyl-diethylenetriamine pentaacetic acid (Gd-EOB-DTPA), a hepatocyte-specific magnetic resonance (MR) contrast agent, is widely used for the detection of focal liver lesions and the characterization of liver tumors.^1–4^ This agent was taken up by hepatocytes after intravenous injection and had a T_1_-shortening effect on normal liver parenchyma, increasing contrast between malignant tumors and normal parenchyma.

MR scanners with a static magnetic field strength of 3.0T were being increasingly used for upper abdominal imaging, and many studies on Gd-EOB-DTPA-enhanced MR imaging using 3.0T equipment have been reported.^5–10^ MR imaging at 3.0T theoretically offered a twofold increase in signal to noise ratio compared with 1.5T,^11,12^ and permits improvement of spatial resolution and/or reduction of the acquisition time. On the other hand, there were potential problems, including more severe specific absorption rate constraints, an increase in imaging artifacts, increased B1 inhomogeneity, and prolonged T_1_ relaxation times.^12,13^ T_1_ contrast may be reduced due to prolongation of T_1_ relaxation times for most abdominal organs at 3.0T compared with 1.5T,^14^ whereas, it has been shown that the T_1_-shortening effects of gadolinium-based contrast agents were relatively unaffected.^15^

In Gd-EOB-DTPA-enhanced MR imaging, sufficient hepatic enhancement on hepatobiliary-phase images, typically acquired 20 min after injection, were required to yield favorable diagnostic performance. The degree of enhancement depends on the function of organic anion-transporting polypeptides of hepatocytes,^16,17^ time after injection,^18,19^ and imaging parameters.^20^ The static magnetic field strength may also influence hepatic enhancement and consequently diagnostic performance.

The liver-to-spleen signal intensity ratio (LSR), the liver-to-muscle signal intensity ratio (LMR), and the contrast enhancement ratio (CER) were widely used to evaluate hepatic enhancement after Gd-EOB-DTPA administration quantitatively.^5,8,10,21^ In this study, we calculated these indices in Gd-EOB-DTPA-enhanced MR images acquired using 1.5T and 3.0T scanners. The principal aim of this study was to determine the effects of static magnetic field strength on hepatic enhancement after Gd-EOB-DTPA administration.

## Materials and Methods

### Patients

This retrospective study was approved by the institutional review board, and the need for informed consent was waived. A total of 40 patients who underwent Gd-EOB-DTPA-enhanced MR imaging between February 2015 and March 2015 were studied. The exclusion criteria were poor breath holding, prior liver resection, and difficulty in appropriate setting of regions of interest (ROIs) in the liver, spleen, or paravertebral muscles. The study subjects were comprised of two groups: the 3.0T and 1.5T groups. In the 3.0T group, initial 20 consecutive patients who were examined on a 3.0T scanner and did not meet the exclusion criteria (10 men and 10 women; 64.5 ± 13.4 years, mean ± standard deviation [SD]) were selected. During this enrollment process, three patients were excluded due to difficulty in appropriate ROI setting (prior liver resection, 1; prior splenectomy, 1; severe atrophy of the paravertebral muscles, 1). A total of 14 of the 20 patients studied, had chronic liver diseases, and hepatic function was classified as Child–Pugh class A in 12 patients and class B in 2 patients. In the 1.5T group, initial 20 consecutive patients who were examined on a 1.5T scanner and did not meet the exclusion criteria (8 men and 12 women; 65.1 ± 10.7 years) were selected. During the enrollment, 1 patient was excluded due to poor breath holding, and 10 patients were excluded due to difficulty in appropriate ROI setting (prior liver resection, 7; severe atrophy of the paravertebral muscles, 2; a large metastatic liver tumor, 1). A total of 13 of the 20 1.5T group patients had chronic liver diseases, and hepatic function was classified as Child–Pugh class A in 10 patients and class B in 3 patients.

### Imaging procedures

Gd-EOB-DTPA-enhanced MR imaging was performed using a 1.5T clinical scanner (Signa HDxt; GE Healthcare, Waukesha, WI, USA) with a 12-channel phased-array coil, or a 3.0T clinical scanner (Discovery 750w; GE Healthcare, Waukesha, WI, USA) with a 32-channel phased-array coil. Our routine imaging protocol included axial in-phase and out-of-phase T_1_-weighted imaging, dynamic imaging using the liver acquisition with volume acceleration (LAVA) sequence, axial and coronal fast spin-echo T_2_-weighted imaging, axial single-shot fast spin-echo T_2_-weighted imaging, axial diffusion-weighted imaging, and hepatobiliary-phase LAVA imaging. Gd-EOB-DTPA (0.025 mmol/kg; Bayer Yakuhin, Osaka, Japan) was administered intravenously in dynamic imaging, and acquisition of hepatobiliary-phase images was started at 20 min after injection. Precontrast and hepatobiliary-phase LAVA images were analyzed for this study.

Precontrast images and post-contrast dynamic images were acquired at a flip angle (FA) of 12°. Hepatobiliary-phase images were obtained at an FA of 12° (low-FA images) and then at an FA of 30° (high-FA images). Typical imaging parameters were field of view = 360 mm, matrix = 320 × 192, slice thickness = 5 mm, and slice number = 44. True spatial resolution was 1.1 × 1.9 × 5.0 mm^3^ and reconstructed spatial resolution was 0.7 × 0.7 × 2.5 mm^3^. Field of view and slice number were increased as required in large patients. A parallel imaging technique (the array spatial sensitivity encoding technique [ASSET]) was used with reduction factors of 2 and 2.5 in the 1.5T and 3.0T scanners, respectively. The receiver bandwidths were ±62.5 and ±83.3 kHz in the 1.5T and 3.0T scanners, respectively. Other imaging parameters are shown in Table 1. For tuning parameters (receiver gain, transmitter gain, center frequency, and gradient shim), the same values were applied to dynamic imaging and low-FA hepatobiliary-phase imaging. Image uniformity correction was performed using phased-array uniformity enhancement (PURE). The preset mode was selected for radiofrequency transmission in the 3.0T scanner.

### Image analysis

**Fig 1.**
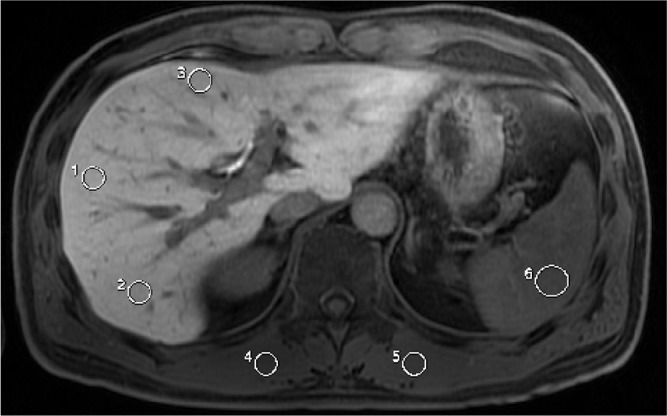
Placement of regions of interest in the liver, muscle, and spleen.

The signal intensity was measured in precontrast images, low-FA hepatobiliary-phase images, and high-FA hepatobiliary-phase images on a picture archiving and communication system (PACS) viewer (EV Insite, PSP Corp., Tokyo, Japan) (Fig. 1). For the liver, 100 mm^2^ circular ROIs were placed in the anterior segment of the right hepatic lobe, the posterior segment of the right lobe, and the medial segment of the left lobe, avoiding vessels, focal liver lesions, and imaging artifacts, on a slice which presented the right main branch of the portal vein. Liver signal intensity was defined as the average of the mean signal intensities in the three ROIs. For muscle, 100 mm^2^ elliptical ROIs were placed in the right and left paravertebral muscles, minimizing inclusion of fat, on the slice that was used to assess liver signals. Muscle signal intensity was defined as the average of the mean signal intensities in the right and left ROIs. A 200 mm^2^ circular ROI was set in the spleen, and spleen signal intensity was defined as the mean signal intensity in the ROI.

Liver signal intensities in the precontrast, low-FA hepatobiliary-phase, and high-FA hepatobiliary-phase images were described as L_Pre_, L_12_, and L_30_, respectively. Muscle signal intensities in the precontrast, low-FA, and high-FA images were designated as M_Pre_, M_12_, and M_30_, respectively. Spleen signal intensities in the precontrast, low-FA, and high-FA images were designated as S_Pre_, S_12_, and S_30_, respectively.

To assess image contrast, the LMR was calculated as the ratio of the liver signal to the muscle signal in the precontrast (LMR_Pre_ = L_Pre_/M_Pre_), low-FA hepatobiliary-phase (LMR_12_ = L_12_/M_12_), and high-FA hepatobiliary-phase (LMR_30_ = L_30_/M_30_) images. The LSR was calculated as the ratio of the liver signal to the spleen signal in the precontrast (LSR_Pre_ = L_Pre_/S_Pre_), low-FA (LSR_12_ = L_12_/S_12_), and high-FA (LSR_30_ = L_30_/S_30_) images. The FA-dependent increase ratio in LMR was calculated as LMR_30_/LMR_12_, and that in LSR was calculated as LSR_30_/LSR_12_. CER was calculated from the liver signal (CER_Liver_ = L_12_/L_Pre_), LMR (CER_LMR_ = LMR_12_/LMR_Pre_), and LSR (CER_LSR_ = LSR_12_/LSR_Pre_) to represent liver signal enhancement. CER was also calculated for muscle (CER_Muscle_ = M_12_/M_Pre_) and the spleen (CER_Spleen_ = S_12_/S_Pre_).

The SD of the liver ROI was divided by the mean signal intensity of the respective ROI and averaged among the three liver ROIs to obtain the liver coefficient of variance (CV). The liver CV was calculated for the precontrast (CV_Pre_), low-FA (CV_12_), and high-FA (CV_30_) images, as indicators of image noise.

## Statistical analysis

The data were presented as means ± SD. Statistical analyses were conducted using R version 2.8.1 (R Foundation for Statistical Computing, Vienna, Austria). Comparisons between the 1.5T and 3.0T groups were made using the unpaired *t*-test. Comparisons between different FAs were made using the paired *t*-test. CER_Liver_, CER_LMR_, and CER_LSR_ were compared for each static field strength using one-way repeated analysis of variance with Bonferroni’s post hoc analysis. Correlation coefficients were compared using the Meng–Rosental–Rubin method. CER_Muscle_ and CER_Spleen_ were compared using the paired *t*-test and the *F*-test. In all analyses, *P* < 0.05 was deemed to indicate statistical significance. A post hoc power analysis was conducted using G*Power version 3.1.9 (University of Duesseldorf, Duesseldorf, Germany).

## Results

When LMRs and LSRs were compared between the 1.5T and 3.0T groups (Table 2), LSR_Pre_ was marginally but significantly smaller in the 3.0T group than in the 1.5T group. There were no significant differences in LMR_Pre_, LMR_12_, LMR_30_, LSR_12_, or LSR_30_, indicating similar image contrast for the 1.5T and 3.0T scanners.

**Fig 2.**
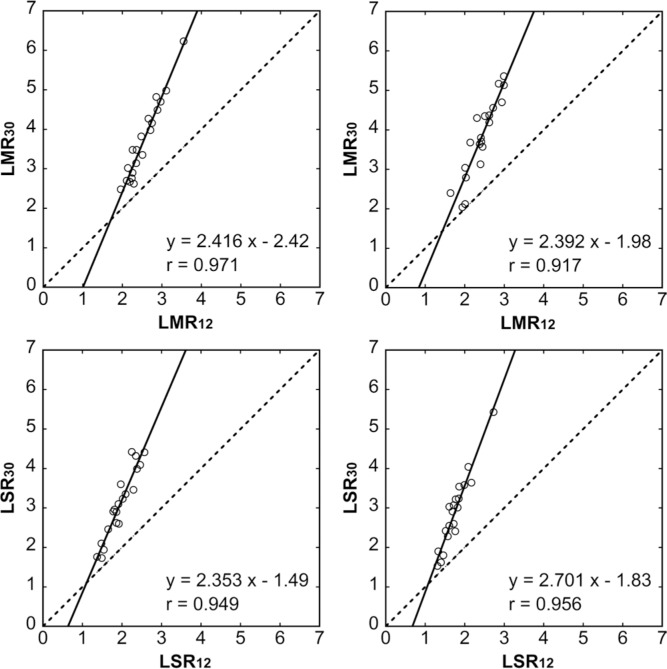
Comparison of the LMR and LSR between different FAs. (**a**) Relationship between LMR_12_ and LMR_30_ at 1.5T. (**b**) Relationship between LMR_12_ and LMR_30_ at 3.0T. (**c**) Relationship between LSR_12_ and LSR_30_ at 1.5T. (**d**) Relationship between LSR_12_ and LSR_30_ at 3.0T. The solid lines represent the regression lines, and the broken lines represent the lines of identity.

In both the 1.5T and 3.0T groups, LMR_30_ was larger than LMR_12_ (*P* < 0.001 in both groups), and LSR_30_ was larger than LSR_12_ (*P* < 0.001 in both groups), indicating higher contrast at a higher FA (Table 2). The FA-dependent increased ratios in the LMR were 1.44 ± 0.17 and 1.55 ± 0.22 in the 1.5T and 3.0T groups, respectively, and those in the LSR were 1.56 ± 0.21 and 1.62 ± 0.25, respectively. There were no significant differences in FA-dependent increased ratios between the groups. There were strong correlations between LMR_12_ and LMR_30_ and between LSR_12_ and LSR_30_ for both 1.5T and 3.0T groups (Fig. 2).

**Fig 3.**
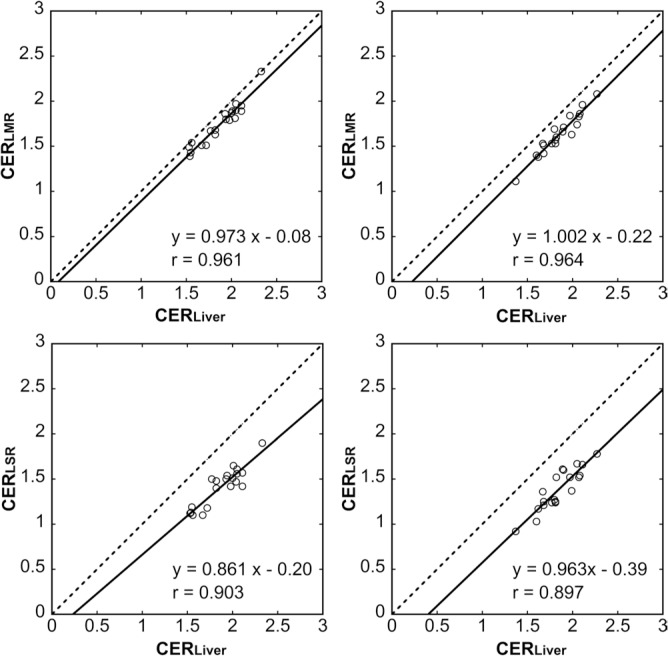
Comparison of CERs calculated using different methods. (**a**) Relationship between CER_Liver_ and CER_LMR_ at 1.5T. (**b**) Relationship between CER_Liver_ and CER_LMR_ at 3.0T. (**c**) Relationship between CER_Liver_ and CER_LSR_ at 1.5T. (**d**) Relationship between CER_Liver_ and CER_LSR_ at 3.0T. The solid lines represent the regression lines, and the broken lines represent the lines of identity.

There were no significant differences in CER_Liver_, CER_LMR_, or CER_LSR_ between the 1.5T and 3.0T groups (Table 3). In both the 1.5T and 3.0T groups, CER_Liver_ was the largest, followed by CER_LMR_ and CER_LSR_. Significant differences were found for all paired comparisons (*P* < 0.001 for all comparisons). Although close correlations with CER_Liver_ were observed for both CER_LMR_ and CER_LSR_ in both the 1.5T and 3.0T groups (Fig. 3), the correlation coefficient was significantly larger for CER_LMR_ (*P* < 0.001 in both groups). CER_Spleen_ (1.5T, 1.33 ± 0.09; 3.0T, 1.35 ± 0.11) was significantly larger than CER_Muscle_ (1.5T, 1.08 ± 0.04; 3.0T, 1.14 ± 0.05) in both the 1.5T and 3.0T groups (*P* < 0.001 in both groups). The SD was also larger for CER_Spleen_ than for CER_Muscle_ (*P* < 0.001 in both groups).

Although CV_Pre_ and CV_12_ were significantly smaller in the 3.0T group than in the 1.5T group, no significant difference was observed for CV_30_ (Table 4).

A post hoc power analysis for the unpaired *t*-test indicated that a total sample size of 40 (1.5T, 20; 3.0T, 20) provided a power of 80% to detect a 0.91 SD difference between groups with a type I error of 5%.

## Discussion

In this study, we quantitatively compared hepatic enhancement in Gd-EOB-DTPA-enhanced MR imaging between 1.5T and 3.0T scanners.

The resulting images were dependent not only on the scanner itself, but also on the parameter settings. It has been reported that the application of high FA values to hepatobiliary-phase imaging increased image contrast and improved lesion conspicuity.^22,23^ In this study, we used two FA values, 12° and 30°. The repetition time and echo time were determined by the scanner software based on the system performance and the specific absorption rate constraints^11,12^ and differed between scanners albeit to small degrees. The receiver bandwidth and the reduction factor of parallel imaging were determined to make the scan time equal between the 1.5T and 3.0T scanners at an FA of 12°. The same receiver bandwidth was used in imaging using different FAs and a given scanner.

This study demonstrated that the LMR and LSR were approximately identical between the 1.5T and 3.0T scanners, which held true for both low and high FAs. The CER was also similar between the 1.5T and 3.0T scanners, irrespective of the definition of CER (CER_Liver_, CER_LMR_, or CER_LSR_). These results indicated that the magnetic field strength does not substantially affect image contrast in Gd-EOB-DTPA-enhanced MR imaging. The lack of dependence on field strength would facilitate inter-scanner comparisons. However, image contrast varied depending on the imaging parameters, and imaging parameters may differ between 1.5T and 3.0T scanners, potentially causing differences in contrast. In addition, the dependence of image contrast on the field strength should be investigated for other manufacturers in future studies.

MR signals were affected by tuning parameters, including receiver gain, transmitter gain, center frequency, and gradient shim. In routine clinical practice, we manually entered the same tuning parameters as the dynamic imaging for hepatobiliary-phase imaging to evaluate hepatic enhancement by direct comparison of the signal intensities. When different tuning parameters were used, it was recommended to evaluate hepatic enhancement using the liver signal normalized to the signal of the reference region, as with CER_LMR_ and CER_LSR_ in this study.^9,10^ CER_LMR_ and CER_LSR_ were correlated with CER_Liver_, supporting their utility for assessment of hepatic enhancement. However, they were significantly lower than CER_Liver_, implying systematic underestimation of contrast effects. This was attributable to increased signal intensity in the muscle and spleen. CER_LMR_ showed less systematic underestimation and better correlation with CER_Liver_ compared with CER_LSR_. Even in the hepatobiliary phase, Gd-EOB-DTPA remained in the blood to various degrees, causing enhancement preferentially in the spleen, a blood-rich organ. The resulting stronger and more variable enhancement of the spleen appeared to explain the larger degree of underestimation and poorer correlation of CER_LSR_.

High image contrast has been reported in hepatobiliary-phase imaging using a high FA.^20,22–25^ In this study, the LSR and LMR in the hepatobiliary phase were larger at a high FA than at a low FA, consistent with the previous reports. The FA-dependent increased ratios in these indicators of image contrast were similar between LSR and LMR and between 1.5T and 3.0T. In addition, there were strong correlations between the contrast indicators obtained at different FAs for both 1.5T and 3.0T. The indices of hepatic enhancement obtained by low-FA imaging have been reported to be correlated with the results of liver function tests.^5,6,21^ Such relationships would also be applicable to indices obtained by high-FA imaging.

In this study, the CV of the liver signal was calculated as an index of image noise. The liver CV was significantly smaller at 3.0T than at 1.5T on low-FA images obtained in both the precontrast and hepatobiliary phases. These observations appeared to be attributed to signal intensification and a consequent increase in the signal to noise ratio at the stronger magnetic field.^11,12^ In contrast, the liver CV was similar between 1.5T and 3.0T in the hepatobiliary-phase high-FA images. Increased magnetic field strength caused prolongation of the T_1_ relaxation time.^11,12^ This prolongation may increase the saturation effect of longitudinal magnetization and decrease the signal to noise ratio. The magnetic field strength was a major but not the sole determinant of the noise property, and depending on the imaging sequence and parameters the noise property may not be improved at 3.0T.

The results of this study had some limitations. First, the study population was relatively small, reducing statistical power, and did not include Child–Pugh class C patients. Second, comparisons between 1.5T and 3.0T were made not only among given patients, but also in different patient groups; and during enrollment to the 1.5T group, many patients were excluded due to prior liver resection. However, there were no obvious differences in patient characteristics between the 1.5T and 3.0T groups, and therefore, our results appeared to reflect the characteristics of 1.5T and 3.0T scanners. Third, there were differences in sequence parameters (repetition time, echo time, receiver bandwidth, and reduction factor of parallel imaging) between the 1.5T and 3.0T scanners. These differences may have affected the results of this study. Fourth, all patients were imaged at an FA of 12° first and then at an FA of 30°. This fixed order may have affected the results of comparison between different FAs; however, the difference in the timing was ∼45 s and the influence appeared to be limited.

## Conclusion

In Gd-EOB-DTPA-enhanced MR imaging, the quantitative indices of hepatic enhancement obtained using 1.5T and 3.0T scanners were similar, indicating that the magnetic field strength does not substantially influence image contrast after Gd-EOB-DTPA administration. This lack of the influence of the field strength appeared to facilitate interscanner comparisons of image findings in clinical practice.

## Figures and Tables

**Table 1.** Scan parameters

Parameter	1.5T		3.0T	
	
	FA = 12°	FA = 30°	FA = 12°	FA = 30°
Repetition time (ms)	4.3	5.6	4.9	5.3
Echo time (ms)	2.0	2.2	1.8	2.0
Flip angle (°)	12	30	12	30
Scan time (s)	16	21	16	15

**Table 2.** Results of the LMR and LSR

Index	1.5T	3.0T	*P*-value
LMR_Pre_	1.45 ± 0.12	1.49 ± 0.17	0.431
LMR_12_	2.53 ± 0.40	2.42 ± 0.38	0.355
LMR_30_	3.70 ± 0.99	3.80 ± 0.99	0.754
LSR_Pre_	1.38 ± 0.14	1.27 ± 0.15	0.026*
LSR_12_	1.95 ± 0.35	1.75 ± 0.33	0.066
LSR_30_	3.10 ± 0.86	2.89 ± 0.92	0.469

Data are presented as means ± SD.

* Statistically significant difference.

**Table 3.** Results of the CER

Index	1.5T	3.0T	*P*-value
CER_Liver_	1.88 ± 0.23	1.85 ± 0.21	0.690
CER_LMR_	1.75 ± 0.23	1.63 ± 0.22	0.115
CER_LSR_	1.42 ± 0.22	1.38 ± 0.23	0.643

Data are presented as means ± SD.

**Table 4.** Results of the CV of the liver signal

Index	1.5T	3.0T	*P*-value
CV_Pre_	5.24 ± 0.97	3.84 ± 0.74	<0.001*
CV_12_	4.19 ± 0.87	3.55 ± 0.96	0.035*
CV_30_	5.30 ± 1.59	5.38 ± 1.50	0.863

Data are presented as means ± SD.

* Statistically significant difference.
